# Forced Oscillation Measurements in Patients with Idiopathic Interstitial Pneumonia Subjected to Pulmonary Rehabilitation

**DOI:** 10.3390/jcm11133657

**Published:** 2022-06-24

**Authors:** Sabina Kostorz-Nosal, Dariusz Jastrzębski, Piotr Kubicki, Dagmara Galle, Alicja Gałeczka-Turkiewicz, Beata Toczylowska, Dariusz Ziora

**Affiliations:** 1 Department of Lung Diseases and Tuberculosis, Faculty of Medical Sciences in Zabrze, Medical University of Silesia, 40-032 Katowice, Poland; djastrzebski@sum.edu.pl (D.J.); piotrkubicki@wp.pl (P.K.); dagmara.galle@gmail.com (D.G.); alicjagaleczka@gmail.com (A.G.-T.); zioradar@wp.pl (D.Z.); 2 Nalecz Institute of Biocybernetics and Biomedical Engineering, Polish Academy of Sciences, 02-109 Warsaw, Poland; beata.toczylowska@ibib.waw.pl

**Keywords:** forced oscillation technique, respiratory oscillometry, pulmonary rehabilitation, idiopathic interstitial pneumonias

## Abstract

(1) Background: Pulmonary rehabilitation (PR) plays a significant therapeutic role for patients with idiopathic interstitial pneumonia (IIP). The study assessed the impact of physical activity on lung function measured by forced oscillation technique (FOT). (2) Methods: The study involved 48 patients with IIP subjected to a 3-week inpatient PR. The control group included IIP patients (n = 44) on a 3-week interval without PR. All patients were assessed at baseline and after 3 weeks of PR by FOT, spirometry, plethysmography, grip strength measurement and the 6-minute walk test. (3) Results: There were no significant changes in FOT measurements in the PR group, except for reduced reactance at 11 Hz, observed in both groups (*p* < 0.05). Patients who completed PR significantly improved their 6-min walk distance (6MWD) and forced vital capacity (FVC). The change in 6MWD was better in patients with higher baseline reactance (*p* = 0.045). (4) Conclusions: Patients with IIP benefit from PR by an increased FVC and 6MWD; however, no improvement in FOT values was noticed. Slow disease progression was observed in the study and control groups, as measured by reduced reactance at 11 Hz. Patients with lower baseline reactance limitations achieve better 6MWD improvement.

## 1. Introduction

Idiopathic interstitial pneumonias (IIPs) constitute a heterogeneous group of noninfectious and noncancerous diseases of unknown etiology [1]. The most common is idiopathic pulmonary fibrosis (IPF), followed by nonspecific interstitial pneumonia (NSIP) and cryptogenic organizing pneumonia (COP). Regardless of the form of the disease, idiopathic interstitial pneumonias are characterized by the presence of diffuse radiological abnormalities in the lungs on high-resolution computed tomography, gradual deterioration into restrictive-type ventilation disorders with reduced diffuse lung capacity and gas exchange impairment [2]. Consequently, patients report the progressive perception of dyspnea, persistent cough and exercise intolerance [1].

Taking into account the progressive character of these diseases and poor response to available treatment options, an extremely important element of comprehensive care for patients with IIP is pulmonary rehabilitation (PR). PR has already proven to have a beneficial influence on the quality of life, dyspnea and exercise capacity in patients with interstitial lung disease (ILD) [3,4,5,6,7,8,9,10,11,12,13,14,15]. According to the National Institute for Health and Care Excellence clinical guidelines [16], PR in IPF patients is recommended every 6–12 months to maintain function in daily living activity, which is impaired in this group of patients [4]. Current evidence indicates greater benefits of PR in ILD patients if administered at the early stage of the disease [10]. Furthermore, desaturation and distance in the 6-minute walk test (6MWT) have been shown to be independent predictors of mortality in IPF patients [17,18]. Therefore, PR, which has already proven to have a positive impact on the 6MWT, plays a central role in patient therapy [3,5,6,7,8,12,15,19].

However, the influence of PR on lung function tests remains unclear. All patients subjected to PR undergo spirometry, which requires forced breathing. By definition, this need for cooperation may cause difficulties in elderly patients (above 65 years old) and children (under 10 years old). For this reason, as an alternative in these groups of patients, it is common to use oscillometry [20,21,22], which has a higher sensitivity than spirometry [23,24,25,26]. According to a recent study [27], oscillometric values provide supplementary data on lung function when compared to more commonly available lung function tests, like spirometry, body plethysmography and diffusing capacity for carbon monoxide.

The forced oscillation technique (FOT), unlike spirometry, is performed during tidal breathing with the application of sound waves into the airways. Through the Fourier transform, it is possible to examine the reaction of the respiratory system to these controlled perturbations. The measured impedance consists of resistance (R) and reactance (X). Resistance reflects the relationship between pressure and the flow of air passing through the airways and is therefore mostly dependent on airway diameter [24,28]. Total resistance at low frequency (at 5 Hz, R5) is determined by sound waves that travel deeply into the small airways. Sound waves at high frequency (at 19 Hz, R19) travel shorter distances and determine the resistance of the central airways. The difference between these two measurements (R5–R19) reflects the small airway resistance. Reactance expresses the ability of the respiratory system to distort [29]. At lower frequencies (X5), it reflects the elastic proprieties of the respiratory system, while at higher frequencies (X19), it reflects its inert properties; lower compliance is expressed as more negative reactance [28]. The difference between inspiratory and expiratory reactance at low frequencies (∆X5) reflects the expiratory flow limitation. Finally, the frequency at which the total reactance is equal to 0 is called the resonant frequency (Fres), which is the result of equality and opposition of the abovementioned elastic and inert properties. Higher values correspond to lower lung compliance.

Recent studies on FOT for ILD patients indicated that small airway disease manifests as reduced X5, increased R5, R5-20, Fres and Ax (area of reactance)—caused by peripheral airway inflammation and fibroproliferation—reduced lung volume and increased elastic recoil pressure in the course of pulmonary fibrosis [30,31,32]. In another study, reactance was indicated to be most affected in interstitial lung diseases [33].

With a view to the characteristic of oscillometry, many researchers had studied the influence of intervention with therapeutic drugs on FOT values in different lung diseases [34]. However, until now, there was only one study analyzing changes in the FOT in chronic obstructive pulmonary disease (COPD) patients subjected to pulmonary rehabilitation [35]. Moreover, there are only a few reports about FOT measurements in IIP patients [30,32,36,37,38]. Therefore, we conducted a prospective cohort study to estimate whether PR has any impact on lung function as measured by FOT. Second, we tried to identify predictors of changes in lung function and exercise tests following PR among the FOT results.

## 2. Materials and Methods

### 2.1. Study Patients

Forty-eight patients with IIP (30 patients with IPF, 9 patients with NSIP and 9 patients with unclassifiable interstitial pneumonia) were recruited between September 2018 and February 2020 to the inpatient rehabilitation program at the Department of Lung Diseases and Tuberculosis, the Medical University of Silesia in Katowice. The control group consisted of 44 patients with IIP (28 patients with IPF and 16 patients with unclassifiable interstitial pneumonia) who did not undergo PR. The study was designed as an intervention non-randomized study with lung function and exercise tests performed before and after 3-week pulmonary rehabilitation/interval. The studied group was created from participants admitted to the pulmonary rehabilitation ward while the control group consisted of corresponding patients admitted to the lung diseases ward or to the hospital out-patient clinic, awaiting pulmonary rehabilitation (the average waiting time in Poland is about one year). Approval was obtained from the Bioethics Committee of the Medical University of Silesia in Katowice (Act No. KNW/0022/KB1/85/I/17 from 19 December 2017) and was conducted in accordance with the Declaration of Helsinki. All patients provided written informed consent. The research was registered in the ISRCTN Trials Registry (ISRCTN31987937). The inclusion criteria were as follows: (1) written informed consent provided by all patients, (2) diagnosis of IIP based on the European Respiratory Society/American Thoracic Society (ERS/ATS) and Polish criteria [1,39,40,41], (3) a stable period of illness without infection/exacerbation during the previous 4 weeks, (4) a distance in 6MWT of over 250 m, (5) and an ability and willingness to perform physical activity.

The exclusion criteria were: (1) patient’s disagreement with the rehabilitation program, (2) infection/exacerbation during the previous 4 weeks, (3) occurrence of connective tissue diseases, (4) unstable coronary artery disease, (5) low performance level (Eastern Cooperative Oncology Group scale ≥ 3), (6) anemia (hemoglobin < 10 g/dL), (7) and poor tolerability of pulmonary rehabilitation.

### 2.2. Physiological Measurements

The primary outcome was the evaluation of FOT results in IIP patients and the influence of PR on these results. The secondary outcomes were the assessment of the other lung function and exercise test results (spirometry, plethysmography and grip strength values and distance and saturation in the 6MWT) and their dependence on PR.

Patients were assessed at baseline and on the last day of the 3-week pulmonary rehabilitation program. The control group underwent FOT, plethysmography and grip strength measurements at baseline and FOT test assessment again after an interval of 3 weeks without pulmonary rehabilitation. Spirometry was performed using a Lungtest apparatus (MES; Cracow, Poland) in accordance with the ATS/ERS guidelines [42]. All results (FEV_1_-forced expiratory volume during the first second, FVC-forced vital capacity, FEV_1_/FVC-forced expiratory volume in one second/forced vital capacity) were expressed as a percentage of predicted values. Plethysmography was measured by a MedGraphic Plethysmograph according to the ATS/ERS guidelines [43], with the results expressed as percentages of the predicted values for total lung capacity (TLC), residual volume (RV) and airway resistance (R_aw_). The grip strength of the left and right hands was evaluated with a Meden-Inmed Baseline hydraulic hand dynamometer according to guidelines [44]. During the test, patients squeezed the dynamometer with all of their hand strength. The maneuver was repeated 3 times with each hand, with the highest value used in the analysis. The single-breath diffusing capacity of the lung for carbon monoxide (TL_CO_) was obtained from the hospital database from an assessment performed up to 3 months before the study by the single-breath method (MedGraphic Plethysmograph), expressed in percentage predicted (TL_CO_ %pred.) according to the ATS/ERS guidelines [45].

The forced oscillation technique was conducted with a Resmon Pro Full device (Restech Respiratory Technology SRL, Italy, Milano; marketed by MGC Diagnostics Cooperation, Saint Paul, MN USA). The measurements were based on the assessment of resistance (R, inspiratory, expiratory and total) at frequencies of 5 Hz, 11 Hz and 19 Hz, reactance (X, inspiratory, expiratory and total) at frequencies of 5 Hz, 11 Hz and 19 Hz, resonant frequency (Fres) and expiratory flow limitation (∆X5). The results were expressed in cmH_2_O/L/s as a percentage of the predicted values and in Hertz for Fres in accordance with Oostveen [46]. The FOT was performed in a sitting position during tidal breathing with the cheeks pressed by the patient’s hands.

### 2.3. Rehabilitation Program

A three-week pulmonary rehabilitation program that conformed to the standard ATS/ERS recommendations [4] was conducted in the hospital under the supervision of a physical therapist. The intensity of training was determined by the limit of the heart rate obtained during the 6MWT. Its program was adapted to the physical abilities of every patient, though every patient had heart rate and blood saturation monitored continuously. The heart rate training in every patient was calculated individually. Pulmonary rehabilitation was carried out at a level not higher than calculated heart rate training and did not result in desaturation under 93% (including patients on long-term oxygen therapy with adjusted oxygen flow rate). In case of the excess over the abovementioned values, a short rest was recommended. During the study, high-intensity rehabilitation was conducted based on the experience of the center [47,48]. PR was held 5 days per week, consisting of endurance, flexibility and resisting training [10], which included exercises on a stabilometric platform (once per day for 20 min), breathing exercises (three times per day for 10 min), lumbar and cervical stabilization exercises and equilibrium exercises (once per day for 20 min), general rehabilitation gymnastics (once per day for 30 min), relaxation (once per day for 30 min) and a cycle ergometer or treadmill (once per day for 30 min in the range of training heart rate).

### 2.4. Statistical Analysis

On the assumption of an effect size equal to 0.5, the group size required to achieve a power equal to 0.99 was 30 cases according to the program G*power. Descriptive statistics are reported as the means with standard deviation. The normality of the distribution was checked using the Shapiro–Wilk test. Factor analysis with the principal component method was performed using varimax normalized rotation. Spearman correlation coefficients were calculated to determine the relationships between measurements. Study/control comparisons at baseline were performed using the *t*-test or Mann–Whitney U test depending on the data distribution. Differences in lung function and exercise test results from baseline to completion of pulmonary rehabilitation were examined by Wilcoxon signed-rank test or the paired test depending on the data distribution. Changes in FOT values after the completion of the pulmonary rehabilitation/3-week interval were assessed by nonparametric analysis of longitudinal data (ANOVA-type statistics, nparLD) [49]. A *p*-value of 0.05 or less was considered statistically significant. The statistical analysis was performed using Statistica 13.3 (TIBCO Software Inc., Palo Alto, CA, USA, License SUM JPZ010A903827ARACD-F).

## 3. Results

Statistical analysis of the demographic parameters within groups and between groups are presented in Table 1. There were differences in the number of male and female participants between the two groups (2 × 2 Fisher test, *p* = 0.01). In the study group, there were 30 male and 18 female (37.5%) patients, and in the control group, there were 38 male and 6 female (13.6%) patients. Because of the small number of female patients, statistical analysis of all parameters was performed for all participants regardless of sex.

Of the total cohort of 48 patients, 54% were treated with antifibrotics (pirfenidone-22 patients, nintedanib-4 patients), 15% were on long-term oxygen therapy, 17% used oral steroids and 25% used bronchodilators (Table 1). Current use of bronchodilators or oral steroids and smoking history for the last 15 years were taken into consideration.

**Figure 1:**
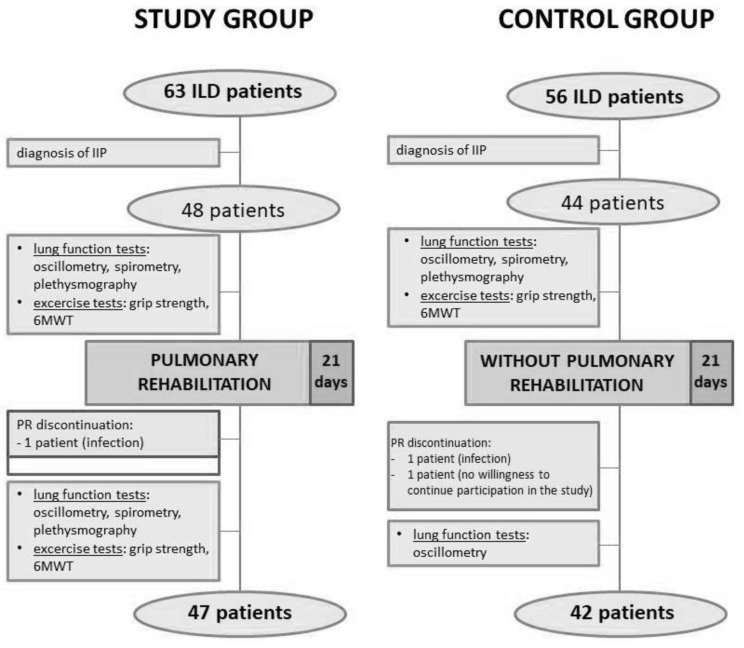
Study recruitment protocol. ILD, interstitial lung disease; IIP, idiopathic interstitial pneumonia; 6MWT, 6-minute walk test; PR, pulmonary rehabilitation.

Forty-seven patients (98%) completed pulmonary rehabilitation and were subjected to a final assessment; one patient discontinued PR due to infection (Figure 1). Of the patients who completed PR, one did not undergo spirometry, two patients were excluded from plethysmography measurements, and six patients were excluded from the 6MWT due to contraindications. Because of the temporary lack of ventilated gas essential for plethysmography, another three patients had missing plethysmographic measurements at the completion of pulmonary rehabilitation. In the control group, two patients were excluded from the study because of infection or lack of willingness to continue participation in the study (Figure 1).

Mean values of lung function and exercise test results at baseline are presented in Table 2. In IIP patients, we observed reduced TL_CO_ and TLC, while the other lung function parameters were mostly preserved. Increased R_aw_ was observed in five patients in the study and control groups, primarily in patients with concomitant obstructive lung disease. There were no significant differences between the lung function and exercise test results between the groups (Table 2).

Among the IIP patients, X was reduced in 46% of the study group patients and 32% of the control group patients, and Fres was increased in 81% and 66% of patients, respectively. In contrast, only 8% of patients presented a higher R, typically those with concomitant obstructive lung disease. Table 3 and Table 4 present analyses of FOT measurements for each patient group.

**Figure 2:**
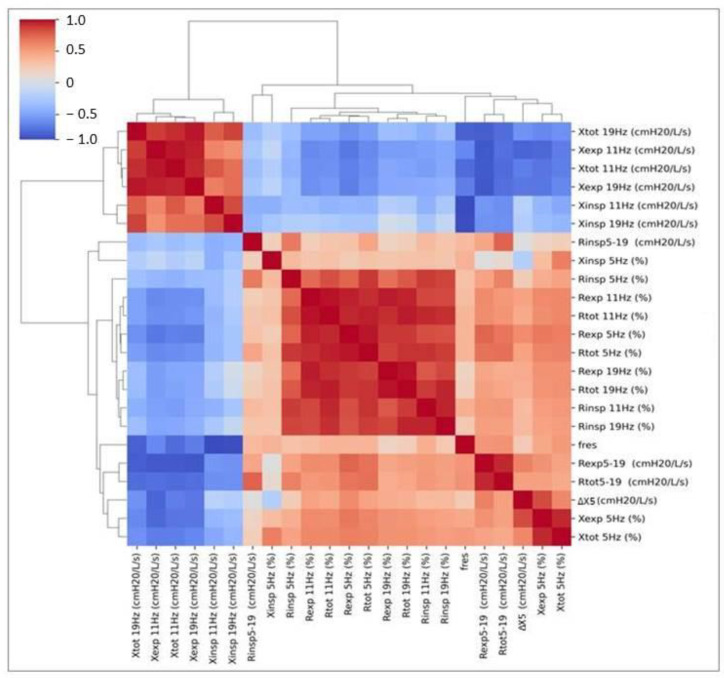
Correlation coefficients between particular resistance and reactance values based on the order of the hierarchical clustering. R, resistance; X, reactance; insp, inspiratory; exp, expiratory; tot, total; ∆X5, expiratory flow limitation; Fres, resonant frequency.

There were strong correlations between particular R and X parameters separately (Figure 2). Increased reactance at a given frequency was accompanied by increased R at other frequencies. Similarly, increased %pred. of X5 or lower measured values of X11 and X19 were accompanied by similar abnormalities at other frequencies. In contrast, increased resistance was observed with higher X5 and lower X11 and X19. In other words, higher abnormalities in R were connected with higher distortions in X.

**Figure 3:**
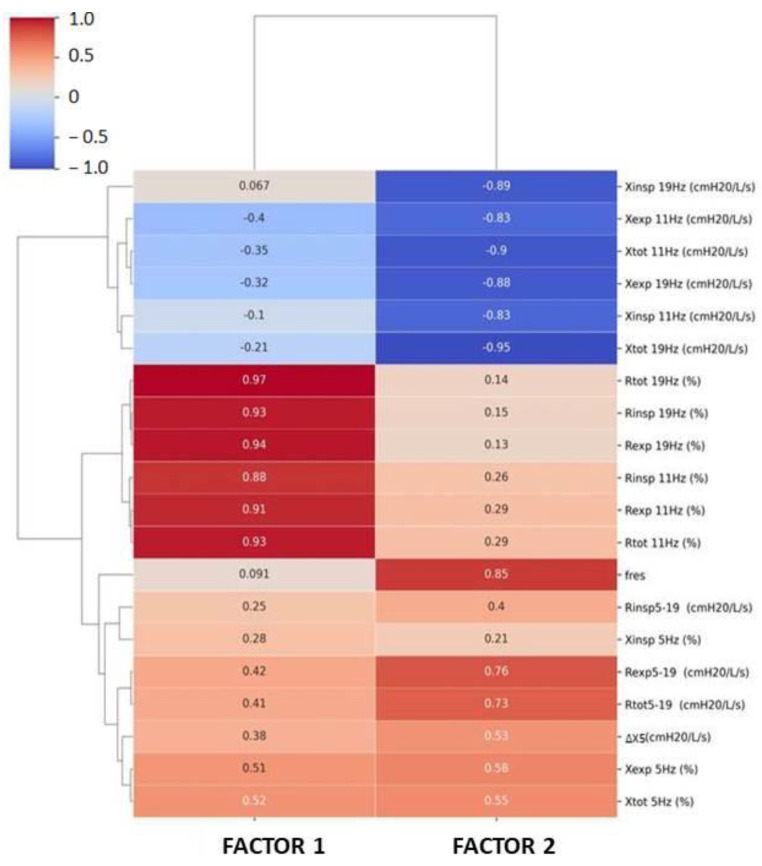
Factor loads obtained by factor analysis of baseline FOT measurements in IIP patients based on the order of hierarchical clustering. R, resistance; X, reactance; insp, inspiratory; exp, expiratory; tot, total; ∆X5, expiratory flow limitation; Fres, resonant frequency.

Factor analysis of baseline FOT measurements in rehabilitated patients with IIP was performed. To maximize the variance, varimax normalized rotation was applied. From all FOT results, we separated two factors (Factors 1 and 2) that reflected 75.3% of the total variance (Table 5). Factor loads are presented in Figure 3. These loads represent correlations between the factor value and individual FOT measurements (a value closer to 1 or −1 corresponds to a stronger positive/negative correlation). It can therefore be concluded that Factor 1 mostly represents resistance values, while Factor 2 reflects reactance and Fres values. Further analysis was performed with the use of the eigenvalues of Factors 1 and 2 (Table 6).

**Figure 4:**
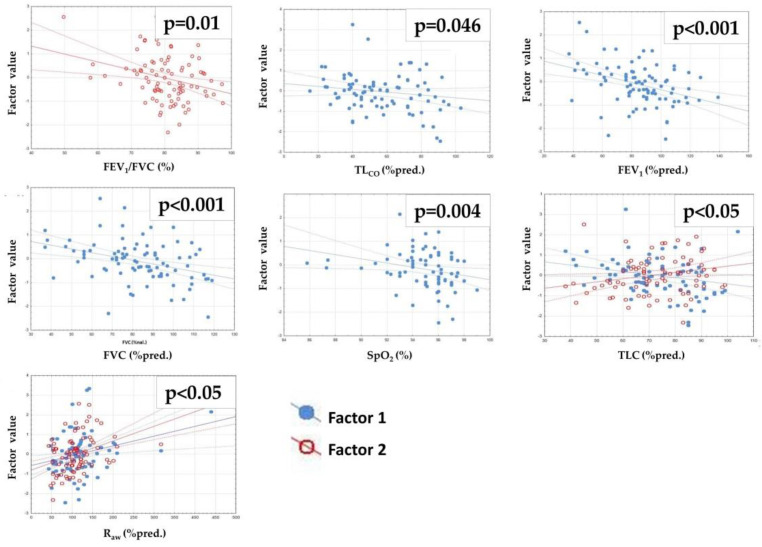
Significant relationships between factors and lung function test results. R, Spearman’s rank correlation coefficient; TL_CO_, lung transfer capacity for carbon monoxide; FEV_1_, forced expiratory volume during the first second; FVC, forced vital capacity; SpO_2_, oxygen saturation; 6MWD, 6-min walk distance; TLC, total lung capacity; RV, residual volume; R_aw_, airway resistance. Factor values were obtained by factor analysis of baseline FOT measurements in rehabilitated patients. Factor 1 reflects mostly resistance values while Factor 2 reflects mostly reactance and Fres values, respectively (see Figure 3).

Table 6 and Figure 4 present correlations between oscillometric factors and other lung function and exercise test results. There was a negative relationship between FEV_1_/FVC and Factor 1 and between Factor 2 and TL_CO_, FEV_1_, FVC and SpO_2_. A positive correlation with Factor 1 and a negative correlation with Factor 2 was observed for TLC. Airway resistance was positively related to both factors.

**Figure 5:**
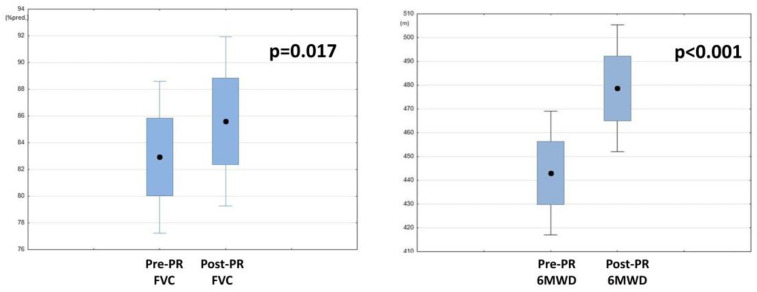
Changes in forced vital capacity (FVC) and 6-min walk distance (6MWD) following pulmonary rehabilitation (PR).

**Figure 6:**
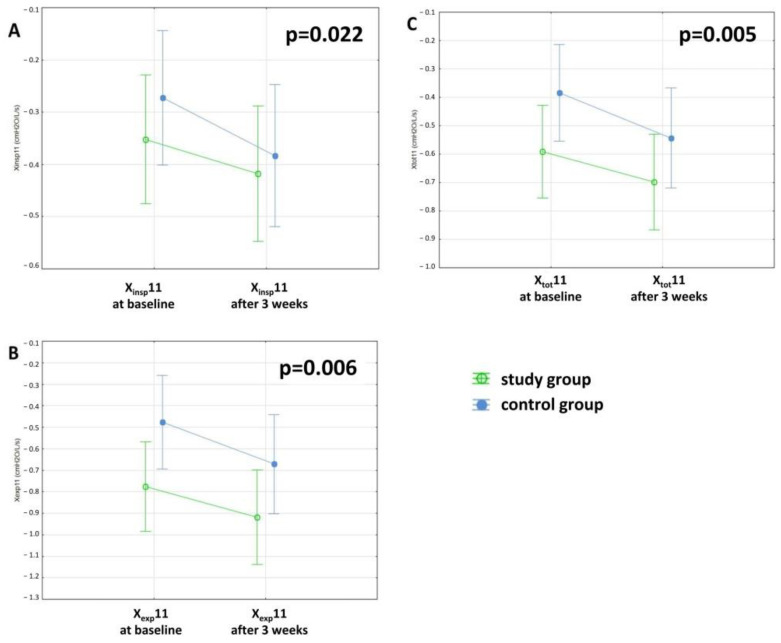
3-week deterioration in reactance at 11 Hz (X11) in the study and control groups, (**A**) inspiratory (insp), (**B**) expiratory (exp) and (**C**) total (tot), respectively.

**Figure 7:**
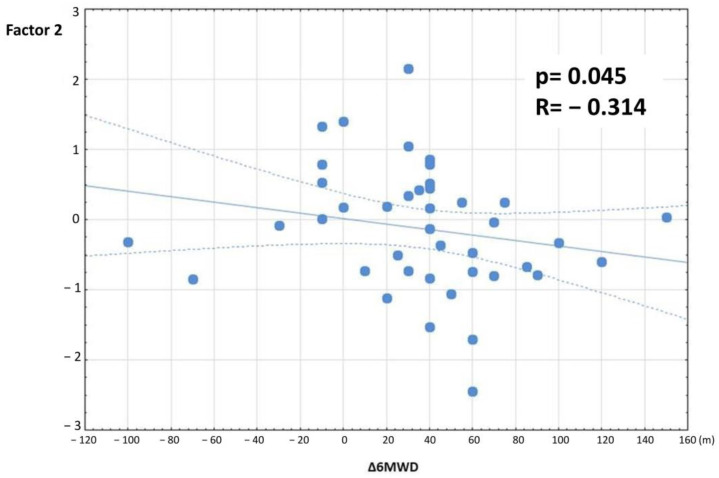
Relationship between Factor 2 and change in the 6MWD (∆6MWD) in the study group. Factor values were obtained by factor analysis of baseline FOT measurements in rehabilitated patients. Factor 2 reflects mostly reactance and Fres values (see Figure 3).

After completing the 3-week PR program, the study group patients demonstrated significant improvements in FVC (*p* = 0.017) and 6MWD (*p* < 0.001, Table 7, Figure 5). Other lung function measurements, including those of the FOT, did not differ, except for X11, which was decreased significantly in both groups (Table 3, Table 4 and Table 7, Figure 6). A strong positive correlation between Factor 2 and change in the 6MWD after PR was observed (Table 8, Figure 7). There was no relationship between the change in FVC and the baseline FOT parameters.

## 4. Discussion

Our study is the first to evaluate the impact of PR on FOT values in patients suffering from IIPs. This is particularly important considering the advanced age at diagnosis of IIPs (for example, for IPF, patients are typically diagnosed at approximately 65 years old) [50], which is related to major problems in performing lung function tests requiring active patient participation. Therefore, the obtained results from spirometry may sometimes be considered unreliable. Additionally, comorbidities may contraindicate forced expiratory maneuvers that are essential in these lung function tests.

However, in statistical analysis, no differences in the FOT parameters were observed following training, while oscillatory measurements showed an important decrease in X11 and a nonsignificant worsening in other X parameters regardless of the use of PR (Table 3 and Table 4, Figure 6). These results are unsurprising, as the patient is aware of the slow progressive course of the disease [9,51,52,53,54]. Although a number of studies have demonstrated an improvement in quality of life after PR [3,5,6,7,8,11,12,13,15,19,55], it is not related to improvements in lung function. More specifically, the discordance between lung function measurements and symptoms that affect the quality of life has already been described [51,56], which explains the occurrence of effects of PR only on the quality of life without any improvements among lung function tests.

In another study [57], we found that disturbances in X11 were more often observed in patients with lung diseases (COPD and IPF, as well as after lobectomy due to lung cancer) than those in X5. Additionally, almost all available studies have focused on X5, without analyzing X11 and X19. In a recent study by Hu et al. [38], only IPF patients with small airway disease (SAD) diagnosed by oscillometric disturbances (not evaluated by spirometry) achieved significant improvement in FEV_1_, FEF_25–75%_ (forced expiratory flow) and symptom scores after bronchodilator treatment. At the same time, IPF patients without SAD had no bronchodilator effect. The thresholds for the bronchodilatory test using FOT parameters are a 40% decrease in R and a 50% increase in X [58]. Similarly in COVID survivors, even when spirometry was normal, changes in oscillometric parameters were detected [59]. The reproducibility of FOT values is estimated to be between 5 and 15% according to the frequency [23]. According to guidelines [24], after proper calibration, a maximum error of 10% is allowed during repeatable measurements. Taking this into account, in total, we observed changes in X by more than 10% in 90% of patients (80 patients). Therefore, the differences observed in our study may be considered significant.

Until now, only one study had analyzed the changes in FOT parameters in COPD patients undergoing PR [35]. In this study, no changes in FOT values following PR were observed except for an improvement in X5, which was maintained at the 3-month follow-up. As X5 reflects airway narrowing and its closure, resulting in increased ventilation heterogeneity, this study emphasized the role of PR in improvements in communicating lung volume rather than changes to lung elastance. These findings are in line with the fact that in COPD patients, increased reactance (X5) is mostly caused by advanced emphysema and therefore hyperinflation [29]. This hypothesis is supported by the findings of the study by Yoshimi [60], who observed a decrease in TLC and RV following PR demonstrating a reduction in hyperinflation. In contrast, in ILD patients, increased reactance is caused by the inability of the lung to distort following increased lung rigidity [29]. These differences may explain the diverse impact of PR on FOT results and the lack of improvement in reactance in IIP patients. Among the possible mechanisms of hyperinflation in COPD patients are the narrowing of the small airways, reduced elastic recoil pressure due to destruction of the alveoli, blood gas abnormalities, and increased chest wall stiffness [61,62,63]. In connection with the above, PR appears to improve mostly lung function reduced by hyperinflation and, to a lesser extent, by fibrosis. In our study, only 6% of patients presented with emphysema.

The conflicts between our study and those of Zimmermann [35] may also be due to the different settings of the rehabilitation program. In the Zimmermann study, COPD patients underwent 16 sessions of two 1 h rehabilitations per week, while our study included 3 weeks of inpatient rehabilitation. Outpatient rehabilitation setting, a more accessible and less cost-consuming method, is not without its difficulties, such as a lack of patient motivation or time for training, unrealistic expectations, lack of physician support and unprofessional relations with the patients [64,65]. However, outpatient rehabilitation in Poland is not commonly used since is not covered by the National Health Fund reimbursement. Salhi et al. [66] suggested that greater improvements could be observed in longer pulmonary rehabilitation programs for patients with restrictive lung diseases lasting 24 weeks. A long-term rehabilitation program may lead to the establishment of a more effective breathing pattern, respiratory muscle strengthening, pleural elasticity and lung compliance improvement [5]. Perhaps a longer duration of PR would have a greater influence on pulmonary function in IIP patients.

Rehabilitation seems to be especially important in IIP patients, given the advanced age of diagnosis. We proved its beneficial impact on the FVC and 6MWD (Table 7, Figure 5). The observed improvement in the 6MWD (*p* < 0.001, mean difference: 35.6 m) is consistent with previous studies [5,7,13,60]. According to Holland [67], for parenchymal lung diseases, a 29–34 m improvement in 6MWD is clinically relevant; in IPF patients, the minimal clinically important distance (MCID) for the 6MWT was calculated to be 24–45 m [68,69]. In another analysis, the MCID for the 6MWT in IPF patients was estimated at 36 m [70]. Similarly, in our study, 49% of patients (23 pts) achieved at least a 36 m improvement in the 6MWD following PR. At the same time, we found better 6MWD improvement in patients whose reactance was less affected at baseline (Table 8, Figure 7). This parameter, which is mostly affected in patients with lung fibrosis, seems to have an essential impact on the effectiveness of PR. These results are in concordance with Zimmermann [35], who showed that a higher limitation in 6MWT was connected with higher spirometric (lower FVC) and FOT disturbances (lower X and higher ∆X). However, Ryerson [8] proved that ILD patients achieved greater improvement in the 6MWD when a worse 6MWD was observed at baseline. This means that even patients with lung function and exercise capacity limitations benefit from PR. To date, there have been no studies on PR predictive factors among FOT results.

Another interesting finding is the significant improvement in FVC (*p* = 0.017, Table 7, Figure 5). There are conflicting reports about the influence of PR on FVC in ILD patients; a number of studies have demonstrated improvements [5,7], while others have demonstrated no impact [12,13,55]. Our study suggests that PR may have a positive impact on FVC. This is extremely important considering the fact that some studies emphasize survival prediction based on a deterioration in FVC [71]. As mentioned earlier, an indispensable part of the course of IIP is the progressive deterioration of lung function, even despite therapy with pirfenidone and nintedanib, which decrease the rate of decline in lung function [9,51,52,53,54]. Therefore, finding a way to prevent FVC worsening appears to be one of the most important elements of complex IIP patient therapy. In this area, PR turns out to be promising.

Chief among the main reasons for exercise intolerance in ILD patients are gas exchange abnormalities, diffusion limitation, peripheral muscle weakness and circulatory limitations [72,73]. All patients included in our study completed 3-week PR, except for one patient who developed a bacterial infection resulting from antibiotic treatment. According to our results, which demonstrated improvements in FVC following PR, there are strong recommendations for future research to define the exact mechanism of lung function improvements in IIP patients after PR.

The question therefore arises: why, in the same group of patients, did we observe significant improvement in FVC and deterioration in X11, given that both the spirometry and oscillometry tests evaluate lung function? Disparities in the influence of PR on these parameters may be explained by the higher sensitivity of oscillometry in detecting SAD [23,24,25]. Second, FOT measurements are performed during tidal breathing, while spirometry requires patient cooperation in forced respiratory maneuvers. This may lead to an improper performance in the first test (with a potential understatement of the FVC at baseline) and significant improvement in the second one [74]. However, in our study, only results that fulfilled the quality criteria for spirometry, including acceptability and repeatability, were included in the study [42]. Third, our PR included breathing exercises, improving diaphragm flexibility and strength. Thus, after PR, patients could have improved breathing maneuvers and achieved better results. Even the mental and quality of life improvements after PR could play an important role in better compliance spirometry performance. Therefore, many factors could have influenced the spirometry results, which simultaneously had no impact on FOT parameters.

Based on factor analysis, we found that Factor 2 was negatively correlated with FVC and TLC and positively correlated with R_aw_ (Table 6, Figure 4). This indicates coherence of these parameters, as lower (more disturbed) reactance and Fres were related to lower FVC and TLC and higher R_aw_. These findings are in concordance with a recent report by Ram et al. [75], who found that resistance, as measured by FOT and with plethysmography, was physically and numerically similar. In contrast, Hellinckx et al. [76] observed that FOT underestimated high resistance values. Concurrently, Meier-Sydow et al. [77] noticed that airway resistance measured by plethysmography was increased in patients with pulmonary fibrosis due to ventilatory inhomogeneity in fibrotic areas and showed a deteriorating tendency with time in one-third of patients. In our study, we did not observe an influence of PR on the plethysmography results.

Our study proved the beneficial impact of PR on IIP patients. We provided a control group that permitted a comparison of the results with those of the study group, revealing deterioration in X11 in both groups, regardless of the use of PR. Moreover, there were no changes regarding the staff or training modality that could have influenced the PR outcomes. Additionally, throughout the duration of the study, all lung function tests were performed by one technician. However, we are aware of the study’s limitations. First, a small number of patients were enrolled in the study. Second, we did not perform a follow-up with an assessment of the long-term effects of PR; therefore, the influence of PR on FOT measurements in the longer term in IIP patients remains unknown. Third, given the sample size, we could not perform a separate analysis of the response to PR for patients with different etiologies and severities of the disease. Moreover, in-hospital stays for rehabilitation in Poland, where the study was carried out, are refunded by the National Health Fund, which, in ILD patients, predicts a maximum in-hospital stay of 3 weeks. Therefore, it was impossible to extend the period of rehabilitation and assess whether the prolonged program could influence FOT parameters. Finally, we could not divide patients into responders and nonresponders based on the improvements in the 6MWD after PR.

In summary, PR does not influence FOT results in IIP patients. Regardless of the type of IIP, these diseases are characterized by a decline in pulmonary function and exercise capacity with time, as reflected in the decline in X11 in both groups. On the other hand, we found possible signs of slowing lung function deterioration due to PR (measured by FVC). Moreover, by improving the 6MWD, PR reduces the risk of mortality in this group of patients [17,18]. Therefore, PR should be considered as an additional therapy in all IIP patients that intends to maintain these components at the highest level possible and thereby ensure patient independence.

## Figures and Tables

**Table 1:** Study demographics.

Parameter	Study Group (48)	Control Group (44)	*p*
Sex	Female: 18 (37.5%)	Female: 6 (13.6%)	0.01 *
	Male: 30 (62.5%)	Male: 38 (86.4%)	
Age (years)	66.0 ± 7.4	66.7 ± 8.5	0.711
BMI (kg/m^2^)	28.3 ± 4.4	28.4 ± 4.3	0.913
Idiopathic interstitial pneumonia type	IPF: 30 NSIP: 9 Unclassifiable IIP: 9	IPF: 28 Unclassifiable IIPs: 16	-
Comorbidities			-
Obstructive lung diseases	3 (6%)	2 (5%)	
Emphysema	3 (6%)	4 (9%)	
Cardiovascular diseases	38 (79%)	31 (70%)	
Diabetes mellitus	6 (13%)	11 (25%)	
Musculoskeletal disorders	26 (54%)	18 (41%)	
Gastrointestinal disorders	14 (29%)	10 (23%)	
Urogenital disorders	15 (31%)	19 (43%)	
Psychiatric disorders	4 (8%)	-	
Smoking history			-
current	1 (2%)	3 (7%)	
previous	24 (50%)	26 (59%)	
never	23 (48%)	15 (34%)	
Antifibrotic treatment	total: 26 (54%)	total: 21 (48%)	-
	pirfenidone: 22 (46%)	pirfenidone: 15 (34%)	
	nintedanib: 4 (8%)	nintedanib: 6 (14%)	
Long-term oxygen therapy	7 (15%)	4 (9%)	-
Oral steroid consumption	8 (17%)	8 (18%)	-
Use of bronchodilator	12 (25%)	6 (14%)	-

* Contingency 2 × 2 Fisher’s test, BMI, body mass index, IPF, idiopathic pulmonary fibrosis; NSIP, nonspecific interstitial pneumonia.

**Table 2:** Lung function and exercise test results at baseline.

	Study Group	Control Group	*p*
TL_CO_ (%pred.)	56.7 ± 20.8	58.7 ± 22.4	0.673 *
FEV_1_ (%pred.)	82.9 ± 20.3	86.6 ± 22.7	0.415 *
FVC (%pred.)	81.5 ± 19.8	84.7 ± 20.5	0.468 *
FEV_1_/FVC (%)	80.9 ± 8.4	80.0 ± 8.3	0.587 ^#^
6MWD (m)	439.6 ± 86.7	463.4 ± 62.0	0.192 *
SpO_2_ (%)	95.1 ± 1.9	94.4 ± 2.9	0.536 ^#^
RV/TLC (%pred.)	106.0 ± 31.6	100.3 ± 22.7	0.366 *
RV (%pred.)	78.5 ± 29.3	75.8 ± 25.8	0.662 *
TLC (%pred.)	73.2 ± 15.7	73.4 ± 13.4	0.955 *
R_aw_ (%pred.)	115.2 ± 69.7	112.9 ± 36.4	0.422 ^#^
RH strength (kg)	32.3 ± 11.9	37.6 ± 10.2	0.052 ^#^
LH strength (kg)	30.1 ± 11.1	34.8 ± 10.0	0.078 ^#^

*—*t* test; ^#^—U-Mann–Whitney test. The results are presented as the mean ± SD. FEV_1_, forced expiratory volume during the first second; FVC, forced vital capacity; SpO_2_, oxygen saturation; 6MWD, 6-min walk distance; TLC, total lung capacity; RV, residual volume; R_aw_, airway resistance; TL_CO_, lung transfer capacity for carbon monoxide; RH, right hand; LH, left hand.

**Table 3:** Resistance (R) at the baseline and after 3 weeks of pulmonary rehabilitation/interval.

Parameter	Study Group		Control Group		nparLD Test *		
	Baseline	After 3-Week PR	Baseline	After 3-Week Interval	Study vs. Control Group (*p*)	Changes in 3-Week in Both Groups (*p*)	Differences in 3-Week Changes between Study and Control Group (*p*)
R_insp_5 (%pred.)	86.29 ± 30.6	84.93 ± 24.7	84.13 ± 26.0	83.92 ± 20.2	0.876	0.329	0.741
R_exp_5 (%pred.)	105.26 ± 36.8	105.07 ± 31.5	96.23 ± 36.5	101.8 ± 41.0	0.28	0.283	0.588
R_tot_5 (%pred.)	97.03 ± 32.0	96.18 ± 26.2	90.8 ± 30.0	94.13 ± 30.5	0.499	0.247	0.518
R_insp_11 (%pred.)	93.78 ± 26.0	91.58 ± 22.0	88.69 ± 24.8	88.44 ± 20.2	0.508	0.916	0.787
R_exp_11 (%pred.)	113.15 ± 30.5	117.26 ± 30.2	105.68 ± 32.5	110.73 ± 37.2	0.249	0.298	0.984
R_tot_11 (%pred.)	104.63 ± 27.2	105.63 ± 25.5	97.98 ± 28.4	101.04 ± 29.2	0.299	0.423	0.868
R_insp_19 (%pred.)	83.03 ± 20.5	82.08 ± 20.4	79.4 ± 22.4	79.35 ± 18.5	0.506	0.905	0.588
R_exp_19 (%pred.)	94.7 ± 21.9	98.4 ± 25.2	90.92 ± 27.2	93.97 ± 29.3	0.383	0.414	0.888
R_tot_19 (%pred.)	89.52 ± 20.2	90.96 ± 22.2	85.71 ± 24.4	87.54 ± 23.9	0.381	0.565	0.869
R_insp_5-19 (cmH_2_O/L/s)	0.29 ± 0.6	0.25 ± 0.6	0.32 ± 0.5	0.31 ± 0.5	0.491	0.843	0.995
R_exp_5-19 (cmH_2_O/L/s)	0.58 ± 0.9	0.42 ± 0.7	0.36 ± 0.6	0.43 ± 0.7	0.853	0.885	0.199
R_tot_5-19 (cmH_2_O/L/s)	0.46 ± 0.6	0.35 ± 0.6	0.35 ± 0.5	0.38 ± 0.5	0.708	0.786	0.427

* ANOVA Nonparametric Analysis of Longitudinal Data. _insp_, inspiratory; _exp_, expiratory; _tot_, total.

**Table 4:** Reactance (X), expiratory flow limitation (∆X) and resonant frequency (Fres) at the baseline and after 3 weeks of pulmonary rehabilitation/interval.

Parameter	Study Group		Control Group		nparLD Test *		
	Baseline	After 3-Week PR	Baseline	After 3-Week Interval	Study vs. Control Group (*p*)	Changes in 3-Week in Both Groups (*p*)	Differences in 3-Week Changes between Study and Control Group (*p*)
X_insp_5 (%pred.)	94.95 ± 42.2	103.09 ± 44.0	102.89 ± 45.8	116.83 ± 66.9	0.378	0.203	0.735
X_exp_5 (%pred.)	115.43 ± 64.8	115.13 ± 59.2	110.83 ± 95.9	117.15 ± 76.3	0.499	0.678	0.475
X_tot_5 (%pred.)	106.78 ± 48.6	110.38 ± 44.8	106.56 ± 61.1	117.21 ± 64.3	0.906	0.349	0.577
X_insp_11 (cmH_2_O/L/s)	−0.36 ± 0.4	−0.42 ± 0.5	−0.28 ± 0.4	−0.38 ± 0.4	0.323	**0.022**	0.402
X_exp_11 (cmH_2_O/L/s)	−0.78 ± 0.9	−0.92 ± 0.9	−0.6 ± 0.9	−0.67 ± 0.6	0.134	**0.006**	0.458
X_tot_11 (cmH_2_O/L/s)	−0.6 ± 0.6	−0.7 ± 0.7	−0.45 ± 0.6	−0.54 ± 0.5	0.172	**0.005**	0.386
X_insp_19 (cmH_2_O/L/s)	0.38 ± 0.4	0.39 ± 0.4	0.37 ± 0.4	0.32 ± 0.4	0.78	0.568	0.517
X_exp_19 (cmH_2_O/L/s)	−0.08 ± 0.6	−0.12 ± 0.6	−0.01 ± 0.6	−0.06 ± 0.4	0.548	0.085	0.578
X_tot_19 (cmH_2_O/L/s)	0.12 ± 0.5	0.11 ± 0.5	0.16 ± 0.5	0.11 ± 0.4	0.808	0.133	0.464
∆X5 (cmH_2_O/L/s)	0.31 ± 0.8	0.19 ± 0.8	0.08 ± 1.1	0.02 ± 0.8	0.092	0.503	0.425
Fres (Hz)	14.96 ± 3.4	15.04 ± 3.4	14.28 ± 4.1	15.26 ± 4.2	0.584	0.095	0.342

* ANOVA Nonparametric Analysis of Longitudinal Data. _insp_, inspiratory; _exp_, expiratory; _tot_, total.

**Table 5:** Proportion of variance explained by factor analysis of baseline FOT measurements.

	% of Variance	Cumulative % of Variance
Factor 1	58.63	58.63
Factor 2	16.67	75.3

Factor values were obtained by factor analysis of baseline FOT measurements in rehabilitated patients. Factor 1 reflects mostly resistance values while Factor 2 reflects mostly reactance and Fres values, respectively (see Figure 3).

**Table 6:** Correlations between factors and other lung function and exercise test results.

		TL_CO_	FEV_1_	FVC	FEV_1_/FVC	Sp0_2_	RV	TLC	R_aw_	6MWD
Factor 1	R p	0.144 0.186	−0.058 0.589	0.012 0.914	**−0.273** **0.01**	0.092 0.438	0.19 0.088	**0.226** **0.042**	**0.398** **<0.001**	−0.158 0.18
Factor 2	R p	**−0.215** **0.046**	**−0.384** **<0.001**	**−0.389** **<0.001**	−0.078 0.468	**−0.333** **0.004**	−0.194 0.081	**−0.318** **0.004**	**0.246** **0.026**	−0.031 0.793

Factor values were obtained by factor analysis of baseline FOT measurements in rehabilitated patients. Factor 1 reflects mostly resistance values while Factor 2 reflects mostly reactance and Fres values, respectively (see Figure 3). R, Spearman’s rank correlation coefficient; TL_CO_, lung transfer capacity for carbon monoxide; FEV_1_, forced expiratory volume during the first second; FVC, forced vital capacity; SpO_2_, oxygen saturation; 6MWD, 6-min walk distance; TLC, total lung capacity; RV, residual volume; R_aw_, airway resistance.

**Table 7:** Lung function and exercise test results in the study group.

	Results at Baseline	Results after 3-Week Rehabilitation	Mean Changes (∆)	95% Cl	*p*
FEV_1_ (%pred.)	82.9 ± 20.3	84.5 ± 20.3	1.4 ± 7.93	−1.05 to 3.84	0.255 *
FVC (%pred.)	82.9 ± 19.8	84.4 ± 21.0	2.67 ± 7.1	0.5 to 4.85	0.017 *
FEV_1_/FVC (%)	80.9 ± 8.4	80.6 ± 7.8	−0.24 ± 4.4	−1.58 to 1.1	0.646 ^#^
6MWD (m)	439.6 ± 86.7	478.7 ± 87.0	35.61 ± 45.5	21.24 to 49.98	<0.001 *
SpO_2_ before 6MWT (%)	95.1 ± 1.9	95.5 ± 2.3	0.32 ± 2.4	−0.45 to 1.09	0.360 ^#^
SpO_2_ after 6MWT (%)	84.5 ± 10.9	86.8 ± 8.7	2.24 ± 7.2	−0.04 to 4.53	0.097 ^#^
RV/TLC (%pred.)	106.0 ± 31.6	103.8 ± 32.3	−2.19 ± 26.7	−10.51 to 6.13	0.644 ^#^
RV (%pred.)	78.5 ± 29.3	78.0 ± 27.2	−0.12 ± 20.2	−6.42 to 6.18	0.970 *
TLC (%pred.)	73.2 ± 15.7	74.5 ± 14.9	1.02 ± 6.6	−1.04 to 3.09	0.323 *
R_aw_ (%pred.)	115.2 ± 69.7	116.5 ± 60.7	12.14 ± 51.4	−3.86 to 28.15	0.255 ^#^
RH strength (kg)	32.3 ± 11.9	32.7± 11.7	0.07 ± 3.5	−0.96 to 1.1	0.397 ^#^
LH strength (kg)	30.1 ± 11.1	31.0 ± 10.2	0.58 ± 3.7	−0.51 to 1.67	0.228 ^#^

*-*t* test; ^#^-Wilcoxon test. All data are presented as the mean ± SD. FEV_1_, forced expiratory volume during the first second; FVC, forced vital capacity; 6MWD, distance in the 6-min walk test; 6MWT, 6-min walk test; SpO_2_, oxygen saturation; TLC, total lung capacity; RV, residual volume; R_aw_, airway resistance; RH, right hand; LH, left hand.

**Table 8:** Correlations between Factors and changes in FVC (∆FVC) and 6MWD (∆6MWD) in the study group.

		∆FVC	∆6MWD
Factor 1	R p	0.033 0.833	−0.064 0.693
Factor 2	R p	0.118 0.452	**−0.314** **0.045**

R, Spearman’s rank correlation coefficient. Factor values were obtained by factor analysis of baseline FOT measurements in rehabilitated patients. Factor 1 reflects mostly resistance values while Factor 2 reflects mostly reactance and Fres values, respectively (see Figure 3).

## Data Availability

Datasets analyzed during the current study are available in the Figshare (https://doi.org/10.6084/m9.figshare.19808377.v1 accessed on 21 May 2022).
